# Establishment and application of a new 4/6 infarct nephrectomy rat model for moderate chronic kidney disease

**DOI:** 10.1590/acb391324

**Published:** 2024-03-11

**Authors:** Kazuhisa Sugai, Momoko Hirano, Asahi Oda, Masahiko Fujisawa, Saori Shono, Katsumi Ishioka, Tomoyoshi Tamura, Yoshinori Katsumata, Motoaki Sano, Eiji Kobayashi, Yoji Hakamata

**Affiliations:** 1 Nippon Veterinary and Life Science University – School of Veterinary Nursing and Technology – Department of Basic Science – Tokyo, Japan.; 2 Nippon Veterinary and Life Science University – School of Veterinary Nursing and Technology – Department of Applied Science – Tokyo, Japan.; 3 Nippon Veterinary and Life Science University – School of Veterinary Nursing and Technology – Department of Veterinary Nursing – Tokyo, Japan.; 4 Keio University – School of Medicine – Department of Emergency and Critical Care Medicine – Tokyo, Japan.; 5 Keio University – School of Medicine – Department of Cardiology – Tokyo, Japan.; 6 Keio University – School of Medicine – Institute for Integrated Sports Medicine – Tokyo, Japan.; 7 Jikei University – School of Medicine – Department of Kidney Regenerative Medicine – Tokyo, Japan.; 8 Nippon Veterinary and Life Science University – Research Center for Animal Life Science – Tokyo, Japan.

**Keywords:** Renal Insufficiency, Chronic, Nephrectomy, Microsurgery, Rats

## Abstract

**Purpose:**

To develop a new 4/6 infarct nephrectomy (INx) model rat mimicking moderate chronic kidney disease (CKD) and to evaluate its application.

**Methods:**

We modified the conventional 5/6 INx rat model to create the 4/6 INx model by ligating the renal artery branch to induce infarction of one-third of the left kidney after right kidney removal and compared biochemically and histologically both models. To demonstrate the application of the 4/6 INx model, the effects of a supplementary compound containing calcium carbonate, chitosan, palm shell activated charcoal etc., that is effective for both CKD and its complications, were compared between both models.

**Results:**

Impairment of renal function in the 4/6 INx group was significantly more moderate than in the 5/6 INx group (*P* < 0.05). The 4/6 INx group showed less histological damage in kidney than in the 5/6 INx group. The supplementary compound did not improve CKD in the 5/6 INx group, but ameliorated elevation of blood urea nitrogen in the 4/6 INx group.

**Conclusions:**

We developed the 4/6 INx model, which is more moderate than the conventional 5/6 INx model. This model could potentially demonstrate the effectiveness of drugs and supplements intended to prevent CKD and its progression.

## Introduction

The kidneys are important organs and have several functions, such as producing urine, regulating blood pressure, maintaining balanced electrolytes, and secreting erythropoietin. Chronic kidney disease (CKD), which refers to long-term impairment of renal function, is a common yet serious disease in human and veterinary medicine^1^^,^ 2. The International Renal Interest Society has developed guidelines that classify CKD in dogs and cats, with classifications from stage 1 to stage 4 based on laboratory values and clinical features^3^. The incidence of CKD in cats particularly increases with age^2^^,^ 4. Although the specific factors that predispose cats to developing kidney diseases are unclear, the resulting decreased renal function prevents the excretion of waste products, which accumulate in the body and cause uremia. Uremia can induce neurological abnormalities that lead to death^5^^–^ 7. Thus, identifying effective treatments for CKD is essential.

In both veterinary practice and human medicine, the use of calorie-controlled diets and specific dietary ingredients are emphasized as approaches to treating CKD and preventing its recurrence and/or progression^8^. Most recently, therapeutic approaches to CKD have focused on use of a low-protein diet combined with effective supplements. Supplementary compounds that have demonstrated isolated beneficial effects include activated charcoal to reduce renal failure by absorbing toxins in the body^9^^,^ 10, *Quercus salicina* (a broadleaf evergreen tree in the Fagaceae family) to prevent formation of uroliths in rats^11^, calcium carbonate to reduce blood phosphate levels in rats with CKD^12^, chitosan (a component of crustacean shells) to alleviate CKD in dogs by absorbing toxins in the intestinal tract^9^^,^ 13 ^,^ 14, sodium alginate to improve renal failure and diabetes when used in combination with chitosan^15^, and folic acid to ameliorate renal anemia and to delay the progression of CKD^16^^,^ 17. However, the effects of supplements in CKD remain unclear, and further basic research is needed.

To date, various genetic models^18^^,^ 19, drug-induced models^20^^,^ 21, and surgical models have been developed to more fully elucidate the pathophysiology of CKD and to aid in developing treatments. Surgical models that achieve decreased renal function by surgically reducing remnant kidney tissue are less time-consuming to produce than other models.

There are two main types of conventional surgical models of CKD. In the 5/6 polectomy model, renal tissue is surgically reduced to decrease renal function^22^. This model involves surgically removing two-thirds of one kidney, followed by total removal of the contralateral kidney after a recovery period, resulting in removal of 5/6 of the total kidney tissue. The 5/6 polectomy model is surgically invasive due to requiring a two-stage operation and has a high mortality rate^21^^,^ 23. The other surgical model of CKD is the 5/6 infarct nephrectomy (INx) model, which induces renal infarction and consequently reduced renal tissue by fully excising one kidney and then ligating the contralateral renal artery branch under a microscope^24^^–^ 26. This model involves a one-stage surgery and has a high survival rate. Both models are estimated to represent approximately 17% of remnant kidney tissue; this corresponds to IRIS stage 3-4 CKD in dogs and cats and a level of CKD that can only be effectively treated by dialysis or kidney transplantation in human medicine.

Prior research initiatives aimed at drug development using 5/6 polectomy and INx models have frequently been abandoned because of a lack of significant therapeutic effect. Given that CKD progresses over time, however, there may be curative opportunities in stage 1-2 CKD before the disease becomes severe. Therefore, we envisioned the need for an animal model that mimics moderate CKD and sought to develop such a model.

In this study, we developed a 4/6 INx model that is a modification of the conventional 5/6 INx model and compared the characteristics of these 2 INx models. To demonstrate the application of the 4/6 INx model, we also compared it to the 5/6 INx model in terms of the demonstrated effects of a supplementary compound (SC) that is considered an effective treatment for both CKD and its associated complications.

## Methods

### Animals

Eight-week-old, male Lewis rats (Charles River Laboratories Japan) were used in the study. The animals were housed in an environment with a room temperature of 23 ± 1°C, humidity of 50 ± 10%, and a 14-hour:10-hour light to dark cycle. They were fed standard chow (CE-2, CLEA Japan, Inc.) ad libitum and allowed free access to water. The rats were acclimatized to this environment for one week prior to any intervention. The animals were randomly divided into the following four groups:

- 4/6 INx (n = 6);
- 4/6 INx SC(+) (n = 6);
- 5/6 INx (n = 6);
- 5/6 INx SC(+) (n = 3).

The animals in the 4/6 INx and 5/6 INx groups were also used as the 4/6 INx SC(-) and the 5/6 INx SC(-) groups, respectively.

The study was approved by the Institutional Animal Care and Use Committee (Nippon Veterinary and Life Science University, Tokyo, Japan; Permit No. 2022K-25), which follows the Nippon Veterinary and Life Science University guidelines for the care and use of laboratory animals. All sections of this report adhere to the ARRIVE guidelines for reporting animal research^27^.

### Surgical procedure for 4/6 and 5/6 INx

**Figure 1:**
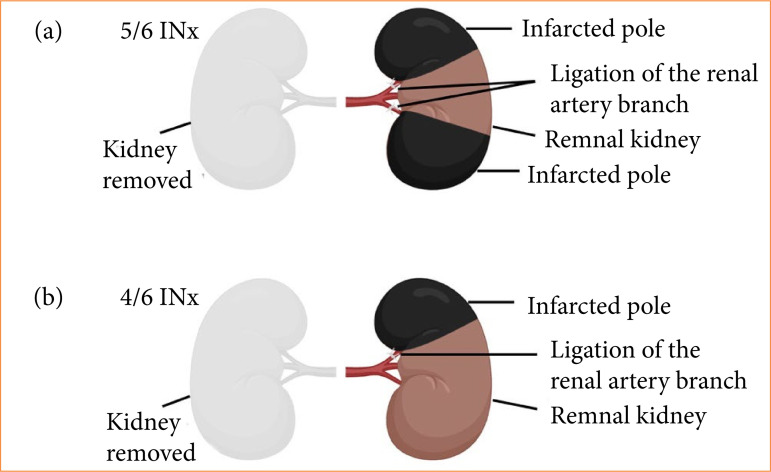
Schema of INx surgery. **(a)** For the 5/6 INx, the right kidney was removed under microscopy; the upper and lower branches of the left renal artery were subsequently selectively ligated to grossly infarct approximately two-thirds of the renal cortex. **(b)** For the 4/6 INx, after removal of the right kidney under microscopy, the upper branch of the left renal artery was selectively ligated to grossly infarct approximately one-third of the renal cortex.

To induce renal failure, 5/6 INx was performed as previously described^26^. In brief, an upper median laparotomy was performed on rats that were under 2% isoflurane inhalation anesthesia. Under a microscope, the right renal artery, renal vein, and ureter were ligated with 4-0 silk sutures, and the right kidney was removed. Subsequently, the upper and lower branches of the left renal artery were identified and selectively ligated with 7-0 silk sutures to grossly infarct approximately two-thirds of the renal cortex. The abdominal wall and skin were closed with 4-0 silk sutures (Fig. 1a).

INx: infarct nephrectomy. Source: Elaborated by the authors.

The 4/6 INx was performed with minor modifications to the 5/6 INx procedure described before. Specifically, the upper branch of the left renal artery was selectively ligated to grossly infarct approximately one-third of the renal cortex (Fig. 1b).

To reduce the surgical invasiveness outside renal infarction, the body temperature of animals was maintained using a heating pad (KN-475-3-40, Natsume Seisakusho Co., Ltd.) during surgery and until fully conscious after surgery. Based on standard veterinary practice, all animals were subcutaneously administered 1-mL/kg antibiotic cefmetazole sodium (0.1 g/mL, NIPRO Co., Ltd.) to the dorsal region of the neck, and were monitored until fully conscious after surgery. The antibiotic was administered once a day for up to two days after surgery.

### Custom diet with supplementary compound for chronic kidney disease

The SC used in this study, which contained calcium carbonate, chitosan, palm shell activated charcoal, *Quercus salicina* extract, sodium alginate, vitamin B^6^, and folic acid, is a dietary supplement for supporting renal function in cats, which was provided by Withpety Co., Ltd. (Kanagawa, Japan). The composition of the SC is shown in Table 1. The SC was mixed into the standard solid diet for rodents (CE-2, CLEA Japan, Inc.) that was provided to the animals, as previously described. The ratio of SC in the diet was adjusted on the basis of the recommended daily feline dose (120 mg/kg body weight) to allow the rats to consume three times the feline dose (360 mg/kg body weight) in anticipation of a loss in intake. Specifically, 3.6 g of SC was added to 1 kg of standard rodent chow. The amount of each component of the SC in a custom diet is shown in Table 1. A custom diet containing the SC was provided to the 4/6 INx SC(+) and 5/6 INx SC(+) groups from postoperative day 1 to day 63.

**Table 1:** Composition of supplementary compound, and the amount of each component of the supplementary compound in custom diet.

Component	Supplementary compound			Custom diet	
	(mg/g SC)	(%)		(mg/g custom diet)	(%)
Calcium carbonate	373.13	37.31		1.34	0.13
Chitosan	373.13	37.31		1.34	0.13
Palm shell activated charcoal	186.84	18.68		0.67	0.067
*Quercus salicina* extract	55.94	5.59		0.20	0.020
Sodium alginate	9.51	0.95		0.034	0.0034
Vitamin B_6_	1.23	0.12		0.0044	0.00044
Folic acid	0.22	0.022		0.00081	0.000081

Source: Elaborated by the authors.

The SC used in this study contained calcium carbonate, chitosan, activated charcoal, *Quercus salicina* extract, sodium alginate, vitamin B_6_, and folic acid. Then, 3.6 g of SC was added to 1 kg of standard rodent chow.

### Blood biochemical and hematological test

A 100-μL blood samples were collected from the jugular vein under 2% isoflurane inhalation anesthesia (as in the previously described surgical procedure), and biochemical tests were performed both before and one, seven, 21, 35, 49, and 63 days after INx procedure. Blood urea nitrogen (BUN), creatinine, hematocrit (Hct), and hemoglobin (Hb) were assessed by using a handheld blood analyzer (i-STAT CHEM8+ cartridge, Abbott Japan Co., Ltd.; i-STAT blood analyzer, Abbott). To quantify the postoperative changes in these biochemical and hematological parameters, change ratios were calculated as the percentage change from the preoperative value.

### Histopathological analysis

On day 63 after INx, the animals were anesthetized with 2% isoflurane (as in the previously described surgical procedure) and euthanized by whole blood collection to facilitate histopathological analysis of the kidneys. The kidneys were removed; kidney specimens were fixed in 10% phosphate-buffered formalin. To evaluate the cortex and medulla from the rostral to the caudal side, fixed tissues were cut longitudinally and embedded in paraffin and then sliced at the maximal cut surface, followed by hematoxylin-eosin and Masson trichrome staining. Each kidney section was scanned with a NanoZoomer-SQ 40× mode (0.23 μm/pixel) scanner (Hamamatsu Photonics K.K.).

The number of glomeruli and the fibrotic area in the entire area of a single section of kidney tissue was quantified by using Fiji/Image J software version 2.9.0/1.53t^28^. The quantified number of remnant glomeruli and fibrotic area were corrected by dividing by the total area of the kidney tissue section and the resulting values were compared between groups.

### Statistical analysis

All data are presented as mean ± standard error of the mean. All statistical analyses were conducted using GraphPad Prism version 9.5.1 (GraphPad Software). Comparisons were performed using a repeated measures two-way analysis of variance (mixed-effects model), multiple unpaired *t* test for post hoc analysis, and unpaired *t* test, as appropriate. A *P*-value < 0.05 was considered statistically significant.

## Results

### Comparisons of the characteristics of 4/6 INx and 5/6 INx models

**Figure 2:**
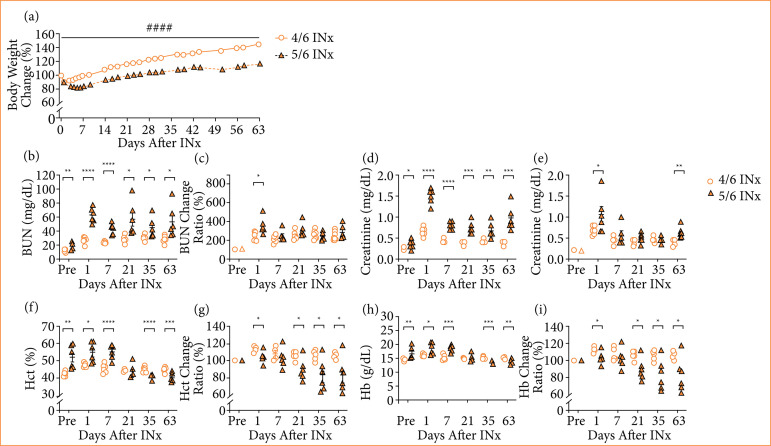
Characteristics of 4/6 INx and 5/6 INx groups. **(a)** Body weight change; **(b)** BUN; **(c)** BUN change ratio; **(d)** creatinine; **(e)** creatinine change ratio; **(f)** Hct; **(g)** Hct change ratio; **(h)** Hb; **(i)** Hb change ratio. Data are expressed as mean ± standard error of the mean. 4/6 INx group, n = 6; 5/6 INx group, n = 6.

BUN: blood urea nitrogen; Hb: hemoglobin; Hct: hematocrit; INx: infarct nephrectomy; Pre: preoperative measurement; *P < 0.05, **P < 0.01, ***P< 0.001, and ****P < 0.0001 for multiple unpaired *t* test; ####*P* < 0.0001 in a repeated measures two-way analysis of variance (mixed-effects model). Source: Elaborated by the authors.

In both the 4/6 INx and 5/6 INx groups, body weight decreased immediately after INx, with a smaller absolute decrease in the 4/6 INx group compared to the 5/6 INx group. Body weight subsequently increased through postoperative day 63 and was significantly higher throughout the entire period in the 4/6 INx group vs. the 5/6 INx group (*P* < 0.0001) (Fig. 2a). Both groups had a 100% survival rate during the experiment.

From the preoperative measurement through postoperative day 63, BUN was significantly lower in the 4/6 INx group compared to the 5/6 INx group (*P* < 0.05) (Fig. 2b). On postoperative day 1, the change ratio for BUN was significantly lower in the 4/6 INx group than in the 5/6 INx group (*P* < 0.05) (Fig. 2c). Similarly, from the preoperative measurement through postoperative day 63, creatinine was significantly lower in the 4/6 INx group compared to the 5/6 INx group (*P*< 0.05) (Fig. 2d). On postoperative day 1 and day 63, the change ratio for creatinine was significantly lower in the 4/6 INx group vs. the 5/6 INx group (*P* < 0.05) (Fig. 2e).

Both Hct and Hb were significantly lower in the 4/6 INx group compared to the 5/6 INx group at the preoperative measurement and on postoperative day 1 and day 7. By contrast, Hct and Hb were significantly higher in the 4/6 INx group vs. the 5/6 INx group on postoperative day 35 and day 63 (*P* < 0.05) (Fig. 2f and 2h). The change ratios for Hct and Hb were significantly higher in the 4/6 INx group than in the 5/6 INx group on postoperative day 1, day 21, day 35, and day 63 (*P* < 0.05) (Fig. 2g and 2i).

### Comparisons of the histopathological changes in 4/6 INx and 5/6 INx models

**Figure 3:**
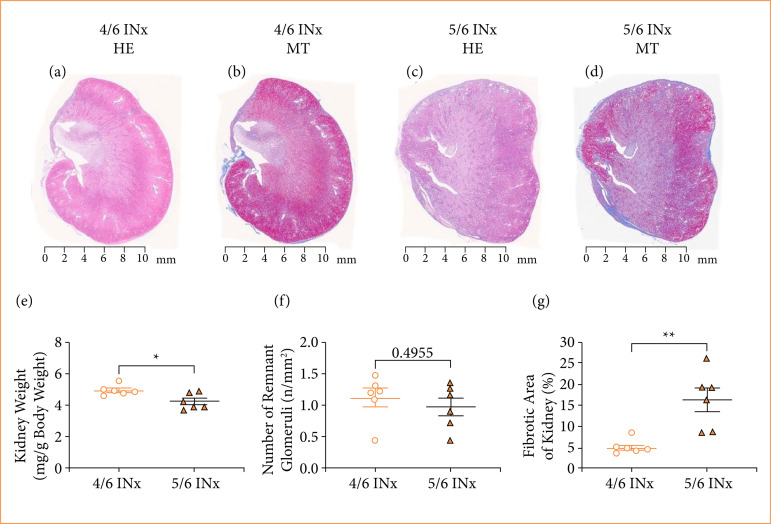
Histopathological changes in 4/6 INx and 5/6 INx groups. **(a)** HE stained kidney in the 4/6 INx group; **(b)** MT stained kidney in the 4/6 INx group; **(c)** HE stained kidney in the 5/6 INx group; **(d)** MT stained kidney in the 5/6 INx group; **(e)** kidney weight; **(f)** the number of remnant glomeruli; **(g)** the fibrotic area of kidney. Data are expressed as the mean ± standard error of the mean. 4/6 INx group, n = 6; 5/6 INx group, n = 6.

HE: hematoxylin-eosin stain; INx: infarct nephrectomy; MT: Masson trichrome stain; *P < 0.05, **P < 0.01 for unpaired *t* test. Source: Elaborated by the authors.

Cortical atrophy was observed in the infarcted areas of the kidneys in both the 4/6 INx and 5/6 INx groups. However, more marked atrophy was seen in the 5/6 INx group compared to the 4/6 INx group (Figs. 3a–3d). Additionally, the kidney weight was significantly lower in the 5/6 INx group than in the 4/6 INx group (*P* < 0.05) (Fig. 3e). The number of remnant glomeruli did not differ between the 4/6 INx and 5/6 INx groups (Fig. 3f). The fibrotic area seen in the 4/6 INx group was significantly smaller than that in the 5/6 INx group (*P* < 0.01) (Fig. 3g).

### Effects of supplementary compound on body weight and renal function in each model

**Figure 4:**
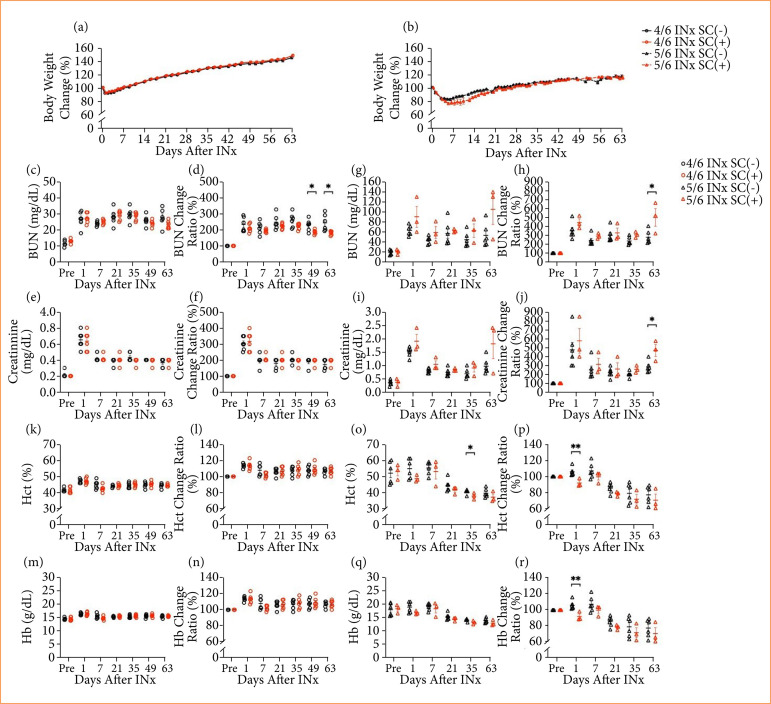
Effects of the SC in the 4/6 INx and 5/6 INx groups. **(a)** Change in body weight in the 4/6 INx SC(-) and 4/6 INx SC(+) groups; **(b)** change in body weight in the 5/6 INx SC(-) and 5/6 INx SC(+) groups; **(c)** BUN in the 4/6 INx SC(-) and 4/6 INx SC(+) groups; **(d)** BUN change ratio in the 4/6 INx SC(-) and 4/6 INx SC(+) groups; **(e)** creatinine in the 4/6 INx SC(-) and 4/6 INx SC(+) groups; **(f)** creatinine change ratio in the 4/6 INx SC(-) and 4/6 INx SC(+) groups; **(g)** BUN in the 5/6 INx SC(-) and 5/6 INx SC(+) groups; **(h)** BUN change ratio in the 5/6 INx SC(-) and 5/6 INx SC(+) groups; **(i)** creatinine in the 5/6 INx SC(-) and 5/6 INx SC(+) groups; **(j)** creatinine change ratio in the 5/6 INx SC(-) and 5/6 INx SC(+) groups; **(k)** Hct in the 4/6 INx SC(-) and 4/6 INx SC(+) groups; **(l)** Hct change ratio in the 4/6 INx SC(-) and 4/6 INx SC(+) groups; **(m)** Hb in the 4/6 INx SC(-) and 4/6 INx SC(+) groups; **(n)** Hb change ratio in the 4/6 INx SC(-) and 4/6 INx SC(+) groups; **(o)** Hct in the 5/6 INx SC(-) and 5/6 INx SC(+) groups; **(p)** Hct change ratio in the 5/6 INx SC(-) and 5/6 INx SC(+) groups; **(q)** Hb in the 5/6 INx SC(-) and 5/6 INx SC(+) groups; **(r)** Hb change ratio in the 5/6 INx SC(-) and 5/6 INx SC(+) groups. Data are expressed as mean ± standard error of the mean. 4/6 INx SC(-) group, n = 6; 4/6 INx SC(+) group, n = 6; 5/6 INx SC(-) group, n = 6; 5/6 INx SC(+) group, n = 3.

BUN: blood urea nitrogen; Hb: hemoglobin; Hct: hematocrit; INx: infarct nephrectomy; SC: supplementary compound; *P < 0.05, **P < 0.01 for multiple unpaired *t* test. Source: Elaborated by the authors.

In both the 4/6 INx and 5/6 INx groups, body weight did not differ depending on whether the animals received a diet with or without SC (Figs. 4a and 4b). In addition, all groups showed a 100% survival rate during the experiment.

On postoperative day 49 and day 63 in the 4/6 INx group, BUN tended to be lower, and the change ratio of BUN was significantly lower among the animals that received the SC(+) diet compared to those on the SC(-) diet (BUN change ratio: *P*< 0.05) (Figs. 4c and 4d). In the 4/6 INx group, the value and change ratio of creatinine did not differ on the basis of whether or not the diet included the SC (Figs. 4e and 4f). From postoperative day 1 through day 63 in the 5/6 INx group, BUN tended to be higher in the animals fed the SC(+) diet compared to those on the SC(-) diet (Fig. 4g). On postoperative day 63 in the 5/6 INx group, the change ratio of BUN was significantly higher among the SC(+) diet group vs. the SC(-) diet group (*P* < 0.05) (Fig. 4h). Similarly, from postoperative day 1 to day 63 in the 5/6 INx group, creatinine tended to be higher among animals fed the SC(+) diet than among those on the SC(-) diet (Fig. 4i). On postoperative day 63 in the 5/6 INx group, the change ratio of creatinine was significantly higher in the SC(+) diet group compared to the SC(-) diet group (*P* < 0.05) (Fig. 4j).

In the 4/6 INx group, the absolute values and change ratios of both Hct and Hb did not differ on the basis of whether animals received a diet with or without the SC (Figs. 4k–4n). On postoperative day 35 in the 5/6 INx group, Hct was significantly lower among animals fed the SC(+) diet compared to those who received the SC(-) diet (*P* < 0.05) (Fig. 4o). On postoperative day 1 in the 5/6 INx group, the change ratio of Hct was significantly lower in the SC(+) diet group than in the SC(-) diet group (*P* < 0.01) (Fig. 4p). In the 5/6 INx group, Hb did not differ between animals fed a diet with or without the SC (Fig. 4q); on postoperative day 1, the change ratio of Hb was significantly lower among animals in the SC(+) diet group vs. those in the SC(-) group (*P* < 0.01) (Fig. 4r).

### Effects of supplementary compound on renal histopathological findings in each model

**Figure 5:**
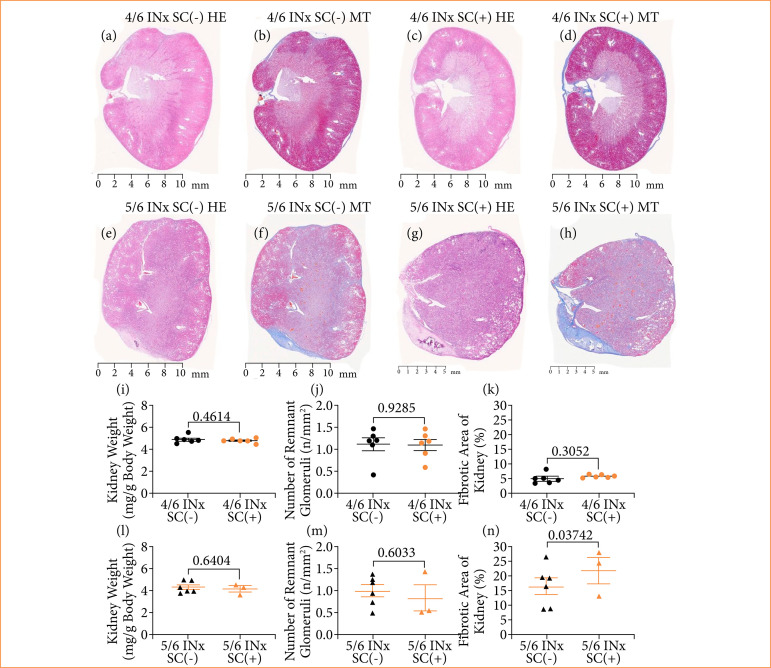
Effects of SC on the kidneys in the 4/6 INx and 5/6 INx groups. **(a)** HE-stained kidney in the 4/6 INx SC(-) group; **(b)** MT-stained kidney in the 4/6 INx SC(-) group; **(c)** HE-stained kidney in the 4/6 INx SC(+) group; **(d)** MT-stained kidney in the 4/6 INx SC(+) group; **(e)** HE-stained kidney in the 5/6 INx SC(-) group; **(f)** MT-stained kidney in the 5/6 INx SC(-) group; **(g)** HE-stained kidney in the 5/6 INx SC(+) group; **(h)** MT-stained kidney in the 5/6 INx SC(+) group; **(i)** kidney weight in the 4/6 INx SC(-) and 4/6 INx SC(+) groups; **(j)** number of remnant glomeruli in the 4/6 INx SC(-) and 4/6 INx SC(+) groups; **(k)** fibrotic area of kidney in the 4/6 INx SC(-) and 4/6 INx SC(+) groups; **(l)** kidney weight in the 5/6 INx SC(-) and 5/6 INx SC(+) groups; **(m)** number of remnant glomeruli in the 5/6 INx SC(-) and 5/6 INx SC(+) groups; **(n)** fibrotic area of kidney in the 5/6 INx SC(-) and 5/6 INx SC(+) groups. Data are expressed as mean ± standard error of the mean. 4/6 INx SC(-) group, n = 6; 4/6 INx SC(+) group, n = 6; 5/6 INx SC(-) group, n = 6; 5/6 INx SC(+) group, n = 3.

HE: hematoxylin-eosin; INx: infarct nephrectomy; MT: Masson trichrome; SC: supplementary compound. Source: Elaborated by the authors.

Both in the 4/6 INx and 5/6 INx groups, cortical atrophy in the infarcted areas of the kidneys did not differ depending on whether the animals received a diet with or without the SC (Figs. 5a–5h). Additionally, the kidney weight, number of remnant glomeruli, and fibrotic area did not differ between animals fed the SC(+) vs. SC(-) diet in either the 4/6 INx or 5/6 INx groups (Figs. 5i–5n).

## Discussion

To date, many CKD models involving surgical techniques have been reported in the pursuit of research to more fully understand this disease state and to develop treatments for CKD^22^^,^ 24 ^–^ 26. However, the 5/6 INx model that has been established already may not be suitable for use in developing therapies for CKD. In long-term experiments, for example, some 5/6 INx model subjects may progress to end-stage kidney disease during the study and thus become ineligible for continued participation to the end of the study^29^. In this study, we developed the 4/6 INx model, which was designed to mimic moderate CKD, by reducing the number of ligated vessels in the renal artery branch compared to the number of vessels ligated in conventional 5/6 INx models. There are no previous reports of CKD models produced using our method.

The 4/6 INx model developed in this study showed less postoperative loss of body weight, faster weight recovery, and better general condition than that seen in the conventional 5/6 INx model. Additionally, renal function declined more moderately in the 4/6 INx group compared to the 5/6 INx group. However, further pathological studies and determination of the glomerular filtration rate and reabsorptive capacity of the kidney are necessary to characterize the 4/6 INx model in more detail. In addition, although the survival rate up to postoperative day 63 was 100% in both models, further investigation is necessary to verify the differences in survival rate between the models after postoperative day 63.

Furthermore, renal anemia, which is a complication of CKD, has been reported in the 5/6 polectomy model^30^^–^ 34. In the 5/6 nephrectomy model induced by renal parenchymal ligation, renal anemia has been reported at five weeks after operation^35^. In this study, renal anemia was also seen in the 5/6 INx group, but not in the 4/6 Inx group, further indicating that the 4/6 Inx model is a moderate CKD model in which renal anemia does not occur. Hct and Hb levels were not affected by extrarenal factors, such as dehydration and intestinal bleeding, and the percentage of remnant normal renal tissue is thus thought to directly reflect renal function. Given these findings, the 4/6 Inx model can be considered a moderate CKD model that corresponds to stage 1-2 CKD in the IRIS guidelines.

To demonstrate the application of 4/6 Inx model rats, a custom diet including a SC was provided to animals in both the 4/6 Inx and 5/6 Inx groups, and the effect of the SC was verified. In the 5/6 Inx group, BUN and creatinine levels were higher in the 5/6 Inx SC(+) group than in the 5/6 INx SC(-) group on postoperative day 63. These phenomena indicate that severe CKD in the 5/6 INx groups progressed, making it impossible to evaluate the effect of the SC. By contrast, a significant decrease in BUN was observed in the 4/6 INx group, demonstrating the effect of the SC. However, the SC did not demonstrate histopathological effect in either model.

Given that activated charcoal, calcium carbonate, and chitosan (all of which were components in the SC) have previously been reported to improve BUN^9^^,^ 10 ^,^ 12 ^–^ 14, and, that the SC was associated with improved BUN in the 4/6 INx SC(+) group in this study, it is possible that continuous intake of the SC may not only affect renal tissue repair, but may also affect protein absorption or metabolic processes outside the kidney, thereby reducing the decline in renal function. Additionally, although the amount of each component contained in the SC was smaller than the quantities used in previous reports^10^^–^ 12 ^,^ 14 ^,^ 15 ^,^ 17 ^,^ 36, the 4/6 INx model’s mimicry of moderate CKD may have allowed the effect of the SC to be apparent.

This study has several limitations. First, although the renal artery branch was ligated to induce infarction in one-third or two-thirds of the renal surface macroscopically, the blood vessel running inside the kidney was not evaluated, and the infarct volume was not precisely assessed. Therefore, the possibility of variation among individuals within both the 4/6 INx and 5/6 INx groups remains. Second, although the efficacy of the SC was demonstrated in the 4/6 INx model, it is unclear which components of the SC were effective. It would be necessary to identify the active ingredients and investigate whether there is a dose-dependent correlation with the observed effect of the SC. Finally, there are reported sex differences in human CKD, with women having a higher incidence of CKD and men being more likely to progress to end-stage kidney disease^37^^–^ 39. However, this study included only male rats. Further studies are needed to determine whether similar results can be reproduced in female rats.

## Conclusion

By selectively ligating the renal artery branch as a modification of the conventional 5/6 INx rat model, we developed a 4/6 INx CKD model that is more moderate and stable than the conventional one. Given the resulting ability to select among different CKD models to suit various specific research purposes, further progress in elucidating this disease state, as well as development of treatments for CKD, can be expected. Particularly, the moderate 4/6 INx CKD model could potentially reveal the effectiveness of drugs and supplements intended to prevent CKD and suppress its progression. The use of different models could also contribute to reducing the number of laboratory animals and improving animal welfare.

## Data Availability

The data will be available upon request.
